# Genotypes of John Cunningham (JC) Virus Urinary Excretion in Pregnant and Non-Pregnant Women in Isfahan, Iran 

**Published:** 2018-06

**Authors:** Sayyedeh Rahmaneh Atyabi, Majid Bouzari, Majid Komijani

**Affiliations:** 1 Department of Biology, Faculty of Sciences, University of Isfahan, Isfahan, Iran; 2 Department of Biology, Faculty of Science, Arak University, Arak, Iran

**Keywords:** John Cunningham Virus, Genotypes, Pregnant Women, Isfahan, Urine

## Abstract

**Objective:** To evaluate presence of different subtypes and genetic variations of JC virus in different geographical areas is a useful tool for reconstructing of the genetic information and understanding of the evolution of the virus and also in tracing of the last and present history of human immigration.

**Materials and methods:** This study aimed to investigate the reactivation of different genotypes of JC virus in kidney and its excretion in the urine of the 50 pregnant and 50 non-pregnant women. Phenol-chloroform method was used to extract DNA. Oligo 7 and MEGA 7 software were used for designing nested PCR specific primers based on *vp1* capsid gene, and construction of phylogenetic tree, respectively. Fisher’s exact test was used for statistical analyses.

**Results:** All of the positive samples were sequenced and according to them, genotypes 1 and 3 of the virus were detected for the first time in pregnant and non-pregnant women in Asia. The frequency of genotypes 1 and 3 were 14.28% and 85.71% respectively.

**Conclusion:** For the first time genotype 3 was reported as the frequent genotype in pregnant women in Asia. Confirming these needs more studies particularly with a higher number of cases and full genome sequencing of isolated JCVs.

## Introduction

Pregnancy is a susceptible period due to changing physiological conditions that can increase susceptibility to external factors such as pathogens, especially opportunistic viruses. Parvovirus B19, lymphocytic choriomeningitis virus (LCMV) and cytomegalovirus (CMV) are well-recognized viruses that have been reported to have adverse consequences on the health of the embryo, fetus, and newborn (1). John Cunningham virus (JCV) is a ubiquitous virus that in 1971 was introduced as the cause of a rare neural disorder named as progressive multifocal leukoencephalopathy (PML) (2). It is reported that the virus may be transmitted through the uterus, sperm, prostate, stool, urine (3), respiratory, perinatal and transplacental routes (4) PML occurs in severely weakened immune system conditions such as application of immunosuppressive drugs for patients with autoimmune diseases, transplant recipients, during infection with HIV and hematological malignancies (2). JCV is classified as *Polyomaviridae* family (4). JCV encodes six viral proteins including large T antigen, small T antigen, Agnoprotein, VP_1_, VP2 and VP_3 _(2). Due to its tumor antigens JCV may combine with high-risk HPV infection in women infected with HIV to influence the rate of progression to invasive cervical carcinoma (5) and breast carcinomas (6) in women. More than ten genotypes of the virus have been detected, which among them, seven types are more important. Genotype 1 is frequent in Europe; types 2 and 7 are frequent in Asia, while frequent genotypes in Africa are types 3 and 6. Type 4 is found in the United States and Europe, which is a recombinant between types, 1 and 3 (2). Different frequencies of urinary excretion of JCV have been reported ranging approximately from 0 to 32.3 percent in pregnant women (7, 8). Genotype 2B is aggressive form of the virus that is reported to be involved in male infertility (3) and PML in immunosuppressed individuals (2). The aim of this study was to detect the frequency of JCV and its different genotypes in the urine of pregnant and non- pregnant women in Isfahan, Iran.

## Materials and methods

***Ethical approval: ***This study was approved by the University of Isfahan ethics committee (No: IR.UI.REC.1396.029). All samplings were performed in compliance with relevant laws and institutional guidelines and in accordance with the ethical standards of the Helsinki Declaration (as revised in Seoul, Republic of Korea, October 2008).

***Urine Samples:*** In a case-control study, urine samples from 50 pregnant women were collected from a reference hospital in Isfahan, during August 2014 – June 2015 and compared with a control group consisted of 50 non-pregnant women. All urine samples were frozen at -20°C until used. 

***DNA Extraction:*** One milliliter of collected urine was centrifuged at 17949×g at 4°C for 10 min. The genome was extracted from 220 microliters of pellet by phenol-chloroform method. In brief, pellet was digested with SDS and proteinase K for 30 minutes at 65°C and genome was extracted with phenol-chloroform-isoamyl alcohol. Then, ethanol and sodium acetate were used for precipitation (9).

***Amplification protocols:*** Human β-globin PCR was performed for determining the accuracy of the extraction (10). Four sets of primers were used for nested PCR for amplifying *vp1* capsid gene. Two sets of primers were used for genotyping (based on the *vp1* gene of Mad-1prototype virus, with accession number: NC_001699) (primers 13-3(5’-ATACATTTGAAAGTGACTCCCC-3’) (first round), 14-4 (5’-TGCCACAGACATCAACAGC-3’) (first round), 13 (5'-GGCCAGAATTCCACTACCC-3') (second round) and 14 (5'-TTCATGACTTGAGATTGCACT-3') (second round)) (11). Confirming the frequency results another two sets of primers (primers 15-5 (5'-CCAGATGAGCATCTTAGGGGTTT-3') (first round), 16-6 (5'-GATTGCACTGTGGCATTCTTTGG-3') (first round), 15 (5'-CAGTGTGGCCAGAATTCCACTACC-3') (second round) and 16 (5'-TAAAGCCTCCCCCCCAACAGAAA-3') (second round)) (12) were used. The PCR reagents used for determining the frequency of the virus used were 20 pmol of each primer, 20 ng DNA, 2.5 µl of 10× Smar Taq DNA polymerase buffer (Tris-HCl 500 mM, Potassium chloride 500 mM, pH= 8.4), 0.3 mM of the dNTP mixture, 1.5 mM MgCl_2_, and 1.5 U of Smar Taq DNA polymerase (CinnaGen, Iran). A volume of 0.5µl of the first round product was used as a template for the second round. For genotyping only *Pfu* Enzyme (Vivantis, Malaysia) was replaced with Smar Taq DNA polymerase and its buffer. The PCRs were performed by an initial denaturation step of 94°C for 5 min, 40 cycles of 94°C for 30 sec, 60°C for 1 min for first round, while this was 64°C for 1 min for the second round, and 72°C for 1 min, with a final extension step at 72°C for 10 min. The PCR products were electrophoresed on agarose gel (1.5%) and then visualized with ethidium bromide under UV light.

***Nucleotide Sequencing:*** All of positive PCR products (using primers 13 and 14 that were designed in this study) were sequenced by Macrogen Corporation (South Korea). Sequences were edited by BioEdit Sequence Alignment Editor Version 7.1.11.0 (13) and compared with already published sequences by using the nucleotide BLAST (National Centre for Biotechnology Information, NCBI). Ten sequences, then, were deposited in the GenBank and DDBJ databases with accession numbers of KT381979, KT381980, KT986209, KT986210, KT986211, KT986212, KT986213, KT986205, KT986206, KT986207 and LC093104. Genotyping was determined by comparison of prototype sequences of known genotypes containing 15 variable nucleotides described in table 2 with detected *vp1* sequences.

***Statistical Analysis:*** Fisher's exact test and GraphPad Prism version 6 (GraphPad USA) software were used for statistical analyses (14).

***Phylogenetic Analysis:*** A phylogenetic tree was constructed in MEGA7 (Molecular Evolutionary Genetic Analysis Software version 7) (15) by using neighbor-joining (NJ) method and 1000 bootstrap replicates.

**Table1:** Frequency of JCV in pregnant and non-pregnant women

**Age groups ** **(years)**	**Pregnant women**		**Non-pregnant women**		**p value**	**OR(95%CI)**
	**Number of ** **urine samples ** **examined**	**Number of JCV ** **positive samples ** **(%)**	**Number of ** **urine samples ** **examined**	**Number of JCV ** **positive samples ** **(%)**		
17-27	23	3(13.04)	19	2(10.52)	1	1.2(0.19-8.54)
28-38	25	4(16)	26	1(3.84)	0.1906	4.76(0.49-45.94)
39-45	2	0)0)	5	1(2)	1	No OR calculated
Total	50	7(14)	50	4(8)	0.5246	1.87(0.51-6.84)

OR: Odd Ratio, CI: Confidence Interval

## Results

***Nested PCR products:*** Expected PCR products of 588 bp and 386 bp were observed using first round primers for detection of different genotypes and frequency of JCV, respectively. In the second round of PCRs, 300 bp and 215 bp products were detected and used for determining the genotypes and frequency of the virus respectively. 

***Frequency of JCV: ***The frequency of JCV in pregnant and non-pregnant women in different age groups is shown in table 1. The virus was detected in 7 out of 50 (14%) of pregnant women and 4 out of 50 (8 %) of non-pregnant women. The difference was not significant (p > 0.05). The differences observed in different age groups were not significant.

***Genotyping of JCV: ***Comparing sequences obtained from pregnant and non-pregnant women with available published sequences in GenBank only two genotypes of 1 and 3 were detected (Table 2). 

**Table 2:** Comparison of JCV Strains in pregnant and non-pregnant women tested in this study with published 15 known polymorphic sites in JCV genome.

**Case**	**Genotypes**	**Nucleotide position in JCV Mad-1 genome (16, 17)**														
		**1** **7** **5** **3**	**1** **7** **5** **6**	**1** **7** **7** **1**	**1** **7** **8** **6**	**1** **7** **9** **0**	**1** **7** **9** **5**	**1** **8** **0** **4**	**1** **8** **0** **5**	**1** **8** **1** **8**	**1** **8** **3** **7**	**1** **8** **4** **3**	**1** **8** **5** **0**	**1** **8** **5** **1**	**1** **8** **6** **9**	**1** **8** **7** **0**
(AF015526) †	1A	A	C	C	G	T	A	T	A	G	T	G	A	C	G	G
(AF015527) †	1B	A	C	C	G	T	A	T	A	G	T	T	G	C	G	G
(AF030085, AF015529, AF015530, AF015531) †	2A	A	C	A	G	T	A	T	A	C	T	T	A	C	G	A
(AF015532, F015533) †	2B	A	C	A	T	T	A	T	A	C	C	T	G	C	G	A
(AF015534-36) †	2C	A	C	A	T	T	A	T	A	C	T	T	G	C	G	A
(U73500, U73502) †	3A	T	C	A	A	T	A	C	A	C	T	T	A	C	C	A
(U73501) †	3B	T	C	A	G	T	A	T	A	C	T	T	A	C	C	G
(AF015528) †	4	A	C	C	G	T	A	T	A	C	T	G	A	C	C	A
(AF015684) †	5	A	C	A	G	C	A	T	A	C	T	T	A	C	G	G
(AF015537) †	6	A	C	A	G	C	A	T	A	C	T	T	A	C	G	G
(U61771) †	7	A	T	A	G	T	G	T	T	C	T	T	A	C	G	A
Samples																
JCVIP1 (KT381979) (P)	3A	T	C	A	A	T	A	C	A	C	T	T	A	C	C	A
JCVIP2 (KT381980) (P)	1A	T *	C	C	G	T	A	T	A	G	T	G	A	C	G	G
JCVIP3 (KT986209) (P)	3A	T	T	A	A	T	A	A	A	C	T	T	A	C	C	A
JCVIP4 (KT986210) (P)	3A	T	C	A	A	T	A	C	A	C	T	T	A	C	C	A
JCVIP5 (KT986211) (P)	3A	T	C	A	A	T	A	C	A	C	T	T	A	C	C	A
JCVIP6 (KT986212) (P)	3A	A	C	A	A	T	A	C	A	C	T	T	A	C	C	A
JCVIP7 (KT986213) (P)	3A	T	C	A	A	T	A	C	A	C	T	T	A	C	C	A
JCVIN5 (LC093104) (NP)	1	A	C	A	N	T	A	T	A	G	T	T	A	C	G	G
JCVIN7 (KT986206) (NP)	3A	T	C	A	A	T	A	C	A	C	T	T	A	C	C	A
JCVIN8 (KT986207) (NP)	3A	T	C	A	A	T	A	C	A	C	T	T	A	C	C	A
JCVIN4 (KT986205) (NP)	3A	T	C	A	A	T	C	A	A	A	T	T	A	A	C	A

N: Not detected, P: Pregnant, NP: Non-pregnant.

* : Underlined nucleotides indicate variation in sequence;

† : Already published JCV strains.

**Table 3:** JCV Genotypes in pregnant and non-pregnant women in Isfahan.

**Samples**	**Number of JCV examined**	**Frequency of different genotypes**	
		**Type 1(%)**	**Type3 (%)**
Pregnant women	7	1(14.28)	6(85.71)
Non-pregnant women	4	1(25)	3(75)
Total	11	2(18.18)	9(81.81)

In comparison with the Mad-1(prototype of JCV) in 15 polymorphic sites that have a major role for genotyping (16, 17), 4 nucleotides differences were observed in isolate JCVIN4. Isolate JCVIP3 showed 2 nucleotides differences, while isolates JCVIP2, JCVIP3 and JCVIN5 showed only 1 nucleotide difference (Table 3). No significant correlation was found among frequencies of JCV types in different age groups in pregnant and non-pregnant women (Tables 2 and 3). Type 3 of JCV as a frequent type, was found in 81.81% (9/11) of the isolates. Type 1 was detected in 18.18% (2/11) of the isolates (Table 3). Among the two subtypes in type 3, only subtype 3A was detected. Notably, type 3 was detected for the first time in pregnant women in the Asia.

**Figure 1:**
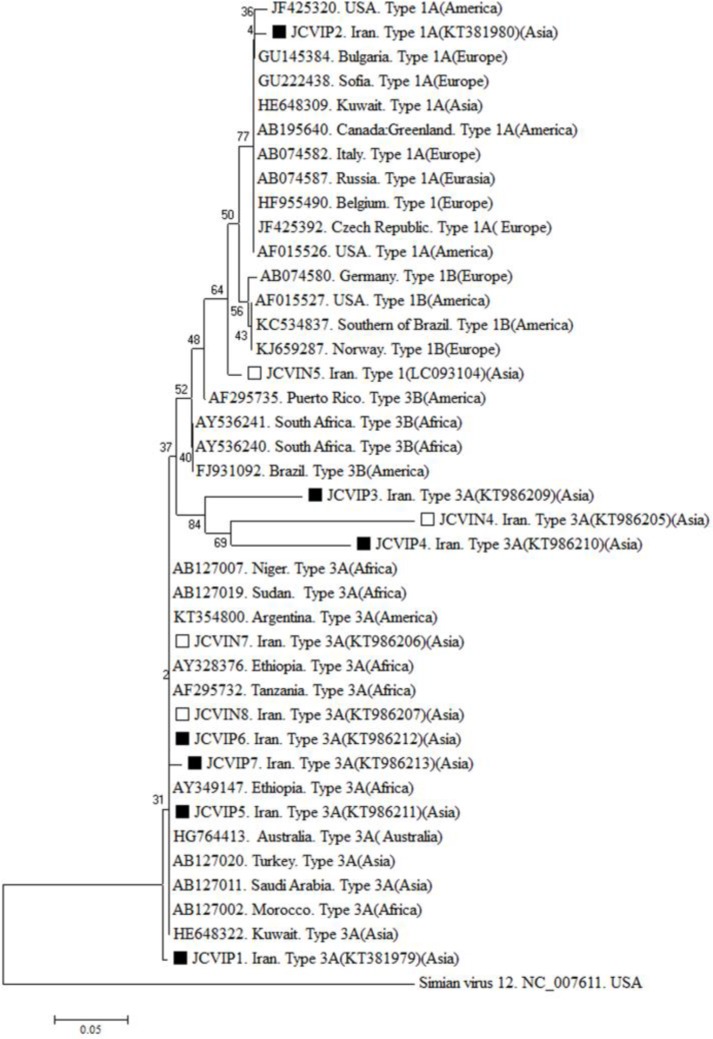
Phylogenetic tree of the detected JCVs in pregnant women and non-pregnant women compared with other countries was constructed using neighbor-joining method implemented in MEGA 7 with 1000 bootstrap replicates. ■: pregnant women, □: non-pregnant women.

***Phylogenetic Analysis:*** A phylogenetic tree was constructed for genotypes 1 and 3 sequences that detected in pregnant and non-pregnant women in this study and the same from different countries obtained from GenBank. Simian virus 12 *vp1* gene was used as an out group (Figure 1). As it is shown in the phylogenetic tree constructed, compare to other sequences, strains JCVIN4, JCVIP3 and JCVIP4 were more similar and were placed in a distinct cluster. Other sequences had higher similarity with the sequences reported from elsewhere.

## Discussion

To retain fetus and preventing abortion by the immune system, the physiology of the body is changed during pregnancy and as a result, pregnant women are more susceptible to pathogens such as viruses (1). JC virus is an opportunistic virus that can be reactivated in immunosuppressed patients and may cause associated disorders as cancer and neurological disorders (2). The aim of this study was to investigate the frequency of the virus and its genotypes in the urine of pregnant and non-pregnant women.

For the first time, in Iran, JCV was detected in the urine of 7 out of 50 (14%) pregnant women cases tested which was less than other reports from Taiwan (32.3%) (7), Norway (0%) (8) and United States of America (22.3% and 7%) (18, 19). No significant difference was observed in the frequency of the virus in pregnant and non-pregnant women which was consistent with other reports from elsewhere. It seems there is no concern for activation of JCV during pregnancy. 

Seven major genotypes have been detected based on *vp1 *capsid gene for JCV. In different geographical areas, genetic variations of JC virus are observed (2). 

According to previous reports based on *vp1* gene, genotypes 4 and 1 are isolated from Europeans and European-Americans. Types 7 and 2 are detected in Asians and Native American populations, while genotypes 2D and 7C are generally found in Asians and south Asians. Genotype 8A is detected in Papua New Guinea, while genotypes 8A, 8B and 2E are found in western pacific populations (2). African-Americans and Africans harbor genotypes 3 and 6. Type 4, as a resulting recombinant of types 1 and 3, is found in Europe and United States (20). Association of PML in immunosuppressed individuals (2) and male infertility (3) are reported for genotype 2B. In this study, types 1 and 3 were detected in 14.28% and 85.71% of the pregnant women for the first time in Iran and Asia, which among them, genotype 3 (subtype A) was frequent. Genotypes 2 and 7 that were already reported as frequent genotypes in Asia were not detected (7). This may indicate the immigration from other areas or they were originally present in the area but not reported.

## Conclusion

Fortunately, aggressive genotype 2B was not found. As it was expected, type 3As detected in this study were very similar and were placed in the same cluster and were very closely related to the sequences reported from Africa, Middle East, South America and Australia. But, type 1 sequences detected were closely related to the sequences reported from the Middle East, North and South America, Eurasia and Europe. This may indicate the origin of different types of the virus detected in this study. Of course, confirming this needs more studies particularly with a higher number of cases and full genome sequencing of isolated JCVs.
